# Does Corporate Social Responsibility Heterogeneity Affect Corporate Financial Performance Through Technological Innovation? The Moderating Effects of Advertising Intensity

**DOI:** 10.3389/fpsyg.2022.837967

**Published:** 2022-07-08

**Authors:** Mengxi Niu, Wentao Ma

**Affiliations:** ^1^Business School, Nankai University, Tianjin, China; ^2^Research School of Accounting, Australian National University, Canberra, ACT, Australia

**Keywords:** technical CSR, institutional CSR, corporate financial performance, advertising intensity, technological innovation

## Abstract

In this study, we examine the effects of firms' corporate social responsibility (CSR), technological innovation, and advertising intensity on corporate financial performance (CFP). Prior research has shown mixed findings for the CSR–CFP relationship. To provide additional evidence and alternative explanations for these mixed findings, we built a moderated mediating model by combining the knowledge-based view with the stakeholder theory. We use this model to examine whether CSR influences CFP by affecting technological innovation, and whether such mediating effects are moderated by advertising intensity. We classify heterogeneous CSR activities into technical and institutional activities. Using data from 2010 to 2018 on Chinese listed firms, we find that superior technical CSR performance can enhance CFP by promoting technological innovation and that it promotes technological innovation to a greater extent when advertising intensity is higher. However, institutional CSR does not affect technological innovation or CFP. The findings suggest that to improve the firm's financial position, its resources should be allocated effectively to technical CSR activities as well as to innovation and advertising.

## Introduction

The term corporate social responsibility (CSR) refers to the efforts of firms to consider social and environmental concerns, and the benefits to their stakeholders, when pursuing business interests (United Nations Industrial Development Organization, 2022). CSR has been a long-standing, yet still crucial, topic in the business world since Bowen (1953) first defined this concept. A firm's financial performance can be influenced by its market and nonmarket strategies (Baron, 2003). CSR, as a major element of such nonmarket strategies, is becoming an instrumental approach to building firms' competitiveness and obtaining resources for their long-term growth (Frynas and Yamahaki, 2016).

Another long-standing discussion, along with that about CSR topics, is whether or not CSR engagement influences corporate financial performance (CFP). Firms consider financial goals their top priority, whereas they often overlook nonfinancial aspirations (e.g., CSR) because conducting CSR activities can incur significant monetary costs, which sometimes draw scarce resources away from their core business activities. Although a stream of literature has examined whether CSR influences CFP, these studies have not arrived at a consensus on this relationship (Waddock and Graves, 1997; Margolis et al., 2009). The CSR–CFP association can be positive (Orlitzky et al., 2003), negative (Wang and Bansal, 2012), absent (Surroca et al., 2010), asymmetric (Van der Laan et al., 2008; Jayachandran et al., 2013), or U-shaped (Barnett and Salomon, 2012), in specific contexts.

The inconsistent findings on the CSR–CFP relationship indicate that further studies need to be conducted to investigate how (mechanisms) and when (contingencies) CSR affects CFP, instead of focusing on the empirical relationship between CSR and CFP alone (McWilliams and Siegel, 2000; Lev et al., 2009). Motivated by this view, in this study, we add technological innovation and advertising intensity, which have largely been overlooked in prior studies, into the CSR–CFP model. Specifically, we examine whether CSR influences CFP through its effects on technological innovation, which is reflected by the investment level for research and development (R&D), and whether such mediating effects are moderated by advertising intensity, which is reflected by the firm's total advertising expenditure. We also take CSR heterogeneity into consideration by separating the integrated CSR performance into the related performance for two specific domains: technical CSR (TCSR) and institutional CSR (ICSR).

Through these analyses, our study makes three main contributions. First, we extend the CSR–CFP literature by providing additional evidence that superior technical CSR performance improves CFP by promoting technological innovation and that this effect tends to be more pronounced as advertising intensity increases (Luo and Du, 2015; Zhao and Murrell, 2016). Further, our analysis results provide mixed findings for the CSR–CFP relationship with an alternative explanation: the effect of CSR performance on CFP also depends on the interaction between the technological innovation and the advertising intensity of firms. Thus, this study responds to the call of Aguinis and Glavas (2012) for deeper insight into the mechanism underlying the CSR–CFP relationship in order to identify effective mediators and moderators.

Second, we distinguish the effects of TCSR from ICSR on technological innovation and CFP. Specifically, we document that only superior TCSR performance can improve CFP by promoting firms' technological innovation. Prior studies generally examine firms' CSR performance by integrating heterogeneous CSR activities into one single construct (Godfrey et al., 2009). However, given that CSR is an “all embracing” idea that includes a wide range of activities related to various stakeholders (Jenkins, 2006, p. 245), the power of tests conducted by combining all CSR-related activities may be limited (Entine, 2003). Our classification of CSR activities allows us to specify how CFP is affected differently by the firm's CSR activities related to its different groups of stakeholders.

Lastly, our study can enhance managers' understanding of the economic consequences of specific CSR activities and the related mechanisms. Our findings indicate to managers that to improve firms' financial position through CSR, they should spend more resources on the activities related to creditors, consumers, shareholders, employees, suppliers, and the government, and that it is critical to promote technological innovation and advertising accordingly.

The remainder of this paper is structured as follows: in the next section, we review a body of relevant literature on the CSR–CFP relationship and on heterogeneous CSR. Thereafter, we develop our hypotheses in Section Hypotheses Development and present our research design in Section Research Design. We describe our sample and report our empirical findings in Section Empirical Results. In Section Conclusion and Discussion, we conclude the study and discuss the findings. Last, in Section Implications and Limitations, we provide this study's theoretical and practical implications and limitations.

## Theoretical Background

### Corporate Social Responsibility and Corporate Financial Performance

In extending the pioneering research regarding the CSR–CFP relationship by Moskowitz (1972), a stream of literature has attempted to identify a clear relationship between CSR performance and CFP. Although the CSR–CFP relationship is inherently a critical strategic topic (Grewatsch and Kleindienst, 2017), to date, this literature has not arrived at a consensus on this relationship. For instance, in a review of more than 170 empirical studies about the CSR–CFP relationship, Rivoli and Waddock (2011) find that these studies have provided mixed findings. Overall, these empirical studies have examined three sets of temporal relationships: positive, negative, and natural CSR–CFP relationships.

In this regard, most studies have supported that CSR can enhance CFP (Dowell et al., 2000; Hillman and Keim, 2001; Orlitzky et al., 2003; Blanco et al., 2013; Servaes and Tamayo, 2013; Wang and Choi, 2013). The positive CSR–CFP relationship is well explained by the stakeholder theory (Freeman, 1984), which emphasizes the importance of fulfilling multilateral stakeholders' expectations, rather than focusing only on bilateral stakeholders' profit maximization aspirations and organizational value creation (Donaldson and Preston, 1995; Jones, 1995). Indeed, CSR promotes rather than limits capital (Hussain et al., 2020), and CSR engagement benefits firms in many ways. For instance, engaging in CSR activities has positive effects on the expansion of firms' market opportunities (King and Lenox, 2009; Pil and Rothenberg, 2009) and their formation of product differentiation (Jones, 1999). CSR engagement can also improve the satisfaction (Edmans, 2012), attraction, retention (Memon et al., 2021), and conscientiousness of employees (Zeng et al., 2020; Yan et al., 2021). Successful CSR engagement is related to positive abnormal returns (Dimson et al., 2015), financial constraint alleviation (Cheng et al., 2014), and increased customer awareness (Servaes and Tamayo, 2013). More broadly, firms that address society's needs benefit because this act builds a favorable reputation and yields them a competitive advantage (Frynas and Yamahaki, 2016).

In contrast, studies that suggest a negative CSR–CFP relationship argue that CSR activities may distract managerial attention and draw resources away from the firm's core business, because managers cannot balance social and financial performance improvement at the same time (Klassen and Whybark, 1999). Schreck (2011) finds no relationship between CSR and CFP. In addition, an asymmetric relationship (Jayachandran et al., 2013) and a U-shaped relationship (Barnett and Salomon, 2012) have been found.

Although the process of exploring empirical relationships is of vital importance for enhancing the understanding and the awareness of CSR, there is still debate and controversy surroundin the ways in which CSR influences CFP (Luo et al., 2015). Given the omitted variable bias (McWilliams and Siegel, 2000), the CSR–CFP relationship is complex and is perhaps more than a direct causal relationship (Margolis and Walsh, 2003). Grewatsch and Kleindienst (2017), who reviewed 32 prominent studies, conclude that the CSR–CFP relationship should be investigated from a contingency perspective. Thus, it is necessary to specify CSR activities and to investigate the mechanism within a specific context to gain additional evidence about the CSR–CFP relationship and to identify practical ways to balance societal concerns and firms' profit-generating activities (Hull and Rothenberg, 2008).

### Corporate Social Responsibility Heterogeneity

The stakeholder concept is an umbrella term for strategic management (Freeman, 1984). As Freeman's metaphor goes, the firm is the hub of a wheel and its stakeholders are at the ends of the spokes around the wheel. Stakeholders who can affect or are affected by the achievement of a firm's purpose (Freeman, 1984) are bound tightly with the firm to achieve a better and more equitable society.

However, the stakeholder concept is a general concept that can be classified into various organizational stakeholder categories. The earliest stakeholder differentiation can be traced back to the period of the depression of the 1930s, for which four main stakeholder groups—shareholders, employees, customers, and the public—have been identified (Preston and Sapienza, 1990). Similarly, shareholders, employees, customers, and managers are included in the strictly business stakeholder group (Clarkson, 1995). Some regular stakeholders, for instance, shareholders, consumers, employees, the community, unions, competitors, suppliers, the government, and the mass media, have also been identified (Freeman, 1984; Sirgy, 2002; Tang and Tang, 2016). In addition, stakeholders are grouped into internal stakeholders (e.g., employees, and business units), lateral stakeholders (e.g., competitors, and the government) (Sirgy, 2002), and external stakeholders (e.g., community, and mass media) (Sirgy, 2002; Tang and Tang, 2018) according to whether they have the same claims and interests, and by the research-specific context. However, the stakeholder groups categorized into primary and secondary stakeholders by Freeman (1984) are more widely discussed (Goodpaster, 1991; Clarkson, 1995; Godfrey et al., 2009). The term primary stakeholders refers to those who possess both the power and the urgency to press their legitimate claims on firms, whereas the term secondary stakeholders refers to those who also have legitimate claims but lack the urgency and the power to enforce their claims (Mitchell et al., 1997; Godfrey et al., 2009). The primary and the secondary stakeholders are also called strategic and moral stakeholders, respectively (Goodpaster, 1991). In a similar vein, Mattingly and Berman (2006) are the first to differentiate CSR activities into TCSR and ICSR, which are aimed at firms' primary and secondary stakeholders, respectively. Godfrey et al. (2009) maintain the idea that CSR includes heterogeneous actions aimed at the different stakeholder recipients of CSR behaviors, and they too classify CSR into TCSR and ICSR.

In line with this literature, we classify CSR into TCSR and ICSR. In our analysis, TCSR is targeted at firms' primary stakeholders, who are essential to business operations and can make legitimate claims on firms since they have both the urgency and the power to enforce those claims. ICSR refers to actions oriented toward secondary stakeholders, who lack the power and the urgency to lodge a claim for CSR activities.

## Hypotheses Development

### Corporate Social Responsibility Heterogeneity and Financial Performance

Given the classification in the previous section, we hold the view that heterogeneous CSR activities may influence CFP through different mechanisms. First, we expect that TCSR is positively associated with CFP. Examples of TCSR activities include improving firms' product quality, employee welfare, or corporate governance, which are somehow a part of firms' normal operational activities that they undertake toward enhancing profitability (Godfrey et al., 2009). TCSR activities involve primary stakeholders, such as customers, creditors, and shareholders. Primary stakeholders can exert strong influence on the firm's operations because their power is utilitarian, coercive, and normative (Mitchell et al., 1997). For example, shareholders can enforce or incentivize management to act in their best interests through management contracts that are decided by the board of directors. Creditors can share information on the creditworthiness of firms, which influences the future financial performance of firms. The exchange capitals of firms are generated by effectively and immediately reacting to the legitimate and urgent claims of primary stakeholders on the firms (Chang et al., 2014). The exchange capitals are reflected in the explicit contracts or the direct exchanges between firms and their stakeholders, such as attracting more high-quality employees, selling more products, reducing material costs, or acquiring more investments (Van der Laan et al., 2008). Therefore, we argue that because primary stakeholders can reward a firm directly by providing financial benefit in exchange for the firm addressing their legitimate and urgent claims, CFP will increase as the firm builds a good relationship with these stakeholders through TCSR activities. Thus, our first hypothesis is as follows:

*H1: TCSR is positively related to CFP*.

Second, we expect that ICSR is also positively related to CFP. ICSR is associated with the legitimate claims by secondary stakeholders who are not directly involved in the firms' operations. ICSR activities include charitable donations and environmental protection activities, which do not align with firms' profit-making interests and are thus unlikely to generate short-term exchange capital to promote CFP, unlike TCSR (Godfrey et al., 2009). Instead, ICSR can increase CFP by creating intangible value, such as a favorable firm reputation.

ICSR allows firms to build a reputation for caring about the well-being of others (Du et al., 2013). Although the reputation *per se* has no cash value, it can generate economic value by influencing the decision-making of these firms' stakeholders because they may perceive that they will also be treated kindly by these firms and thus tend to make decisions that are favorable for the firms. Thus, ICSR activities have been found to improve employee commitment (Brammer and Millington, 2005), firms' attractiveness to high-quality employees (Jones et al., 2014), and customer satisfaction with the brand (Lee et al., 2009). Consequently, CFP will increase with the increase in productivity and sales. Thus, our second hypothesis is as follows:

*H2: ICSR is positively related to CFP*

### Technological Innovation

The knowledge-based view sheds light on the importance of knowledge in firms. The knowledge that is created, stored, and exploited within firms can be regarded as an important strategic resource to drive business development (Grant, 1996). Hakanson (2010) argues that as social entities, firms should pay more attention to their in-house knowledge storage and application, which are the determinants for the survival and the future development of firms.

The acquisition and application of firm knowledge, in the form of technological innovation, can improve CFP because technological innovation facilitates product and process renewal, which, in turn, improves productivity (McWilliams and Siegel, 2000). Technological innovation, as a part of firms' core capabilities, also allows firms to meet dynamic market requirements by developing products of superior quality and thus improve their profitability (Cassiman and Veugelers, 2006).

Meanwhile, according to the knowledge-based view, technological innovation can be promoted by stakeholder-oriented CSR activities (Luo and Du, 2015). Favorable stakeholder relationships boost firms' access to valuable information (Harrison et al., 2010; Desai, 2018). Effective knowledge transformation and mutual learning with stakeholders facilitate knowledge recombination and the access to resources needed for firms' innovation success (Jiang et al., 2020).

Taken together, we argue that technological innovation can be a key channel for CSR to create business value. Stakeholders are more likely to share their knowledge with firms who broaden and deepen their relationships with the stakeholders through CSR engagement (Luo and Du, 2015). Compared with the existing knowledge of firms, the incremental knowledge they can acquire from stakeholders is quite novel. This novel knowledge as well as the resources and the support from stakeholders facilitates innovation and thus ultimately improves CFP (Jiang et al., 2020).

Moreover, Hull and Rothenberg (2008) highlight that heterogeneous CSR activities may lead to different CSR–CFP relationships, and the interactions between differentiated CSR activities and technological innovation can also vary. Therefore, it is worth exploring the CSR–CFP relationship according to the different mechanisms between CSR heterogeneity and technological innovation. In this study, we deepen our analysis by identifying the comprehensive relationship between CSR heterogeneity, technological innovation, and CFP. In general, although we infer that TCSR and ICSR activities may both improve CFP, TCSR and ICSR activities play different roles in driving technological innovation and CFP. Specifically, we predict that the mediating effect of technological innovation in the CSR–CFP relationship can be valid only when firms care for their primary stakeholders; that is, technological innovation mediates only the TCSR–CFP relationship and not the ICSR–CSP relationship. TCSR activities target the primary stakeholders, whereas ICSR activities target the secondary ones. Unlike secondary stakeholders, primary stakeholders can involve themselves in firms' economic transactions directly, and hence, their claims are more authoritative, legitimate, and urgent than those of the secondary stakeholders. Moreover, firms find it easier to build extensive and close networks with their primary stakeholders. Knowledge exchanges are thus more likely to be conducted in the interactions between firms and their primary stakeholders. As Thompson and Heron (2006) indicate, the relationships between firms and their primary stakeholders can be regarded as a certain relational capital, and the quality of this capital significantly affects firms' innovative abilities. By contrast, the secondary stakeholders' claims on firms always lack adequate support, because they hardly contribute any human capital or other valuable resources to firms but lead the firms to bear the risks in business operations alone (Mitchell et al., 1997). Hence, it is difficult for a firm to establish stable relationships with its secondary stakeholders, and the firm cannot foster technological innovation through knowledge exchange with them. Therefore, we propose that TCSR and ICSR can influence CFP differently:

*H3: Technological innovation mediates the relationship between TCSR and CFP; that is, TCSR improves CFP through technological innovation*.
*H4: Technological innovation does not mediate the relationship between ICSR and CFP; that is, ICSR cannot improve CFP through technological innovation*.

### Advertising Intensity

Advertising, which is a strategic marketing lever and a market-based maker of intangible assets, is widely applied in business competition (Luo and Bhattacharya, 2009). The term advertising intensity refers to the total advertising expenditure relative to a firm's overall resources (Huang and Wei, 2012). Advertising intensity provides a firm-specific context in examining the CSR–CFP relationship (McWilliams and Siegel, 2000). First, more effective advertising tactics and more advertising expenditure improve firm product differentiation (Bain, 1956). Incumbent firms can take advantage of advertising to establish and maintain a monopoly and thus erect competitive barriers and create competitiveness in business, which helps these firms to overcome potential risks in competing with new entrants (McGee, 1988). For new entrants, increased advertising investments can assist them to offset a disadvantage, namely, that the incumbent firms have already gained brand recognition among customers (Robinson and McDougall, 2001). Second, advertising intensity leads to a mitigation of information asymmetry (Rahman et al., 2017). Sufficient product information facilitates customer decision-making as regards purchasing those products (Nelson, 1991). Advertising is used to implement a competitive strategy against the firm's competitors Advertising is a means of business competitive strategy among firm competitors (Scherer and Ross, 1990) because firms can use advertising not only to promote products and services but also to persuade customers that the advertised product is superior to its counterparts. A high level of advertising investment by a firm makes it easier for customers to obtain a unique image of this firm, which effectively mitigates reputation asymmetries between firms and their target customers (Shamsie, 2003). In addition, advertising can increase the potential demand for firm products by reducing the search costs of those latent customers; thus, advertising is a critical tool in gathering externality (Stahl, 1982).

CSR makes an impact by delivering the information that is expected to differentiate firms upward from their competitors (Schuler and Cording, 2006; Mackey et al., 2007). For example, customers are more willing to purchase products from socially reputable firms (Rahman et al., 2017) because CSR information highlights firm' organization-based self-esteem and creates positive moral capital. Advertising intensity can significantly increase the overall amount of information available to stakeholders, which may either positively or negatively impress them (Servaes and Tamayo, 2013). Taken together, we argue that CSR promotes technological innovation by impressing stakeholders with positive information, whereas the information from other sources, such as advertising, may positively or negatively moderate the impact of CSR-related information (Schuler and Cording, 2006).

In line with the knowledge-based view, one possible inference is that advertising intensity and TCSR have a synergistic effect in the relationship between TCSR and technological innovation. Advertising intensity may strengthen the positive relationship between TCSR and technological innovation because a good reputation generated from high advertising intensity strengthens knowledge sharing between primary stakeholders and firms, and, in turn, improves firms' technological innovation.

However, there may be a substitutional relationship between advertising intensity and TCSR in that advertising intensity may weaken the positive relationship between TCSR and technological innovation. Advertising significantly influences the accumulation process of firm reputation and brand loyalty. The high uncertainty, high asset specificity, and high sunk costs of the process exacerbate the significant constraints, which are caused by scale economy, resource erosion, and time compression diseconomies, on knowledge acquisition through CSR activities. For example, offensive advertising may impress stakeholders negatively, or too much advertising may distract stakeholder attention from the firm's CSR-related information. Therefore, we develop two competing hypotheses:

*H5a: There is a synergistic interaction between advertising intensity and TCSR. Specifically, the higher the advertising intensity is, the stronger is the positive impact on TCSR and technological innovation relationship*.
*H5b: There is a substitutional interaction between advertising intensity and TCSR. Specifically, the higher the advertising intensity is, the weaker is the positive impact on TCSR and technological innovation relationship*.

**Figure 1:**
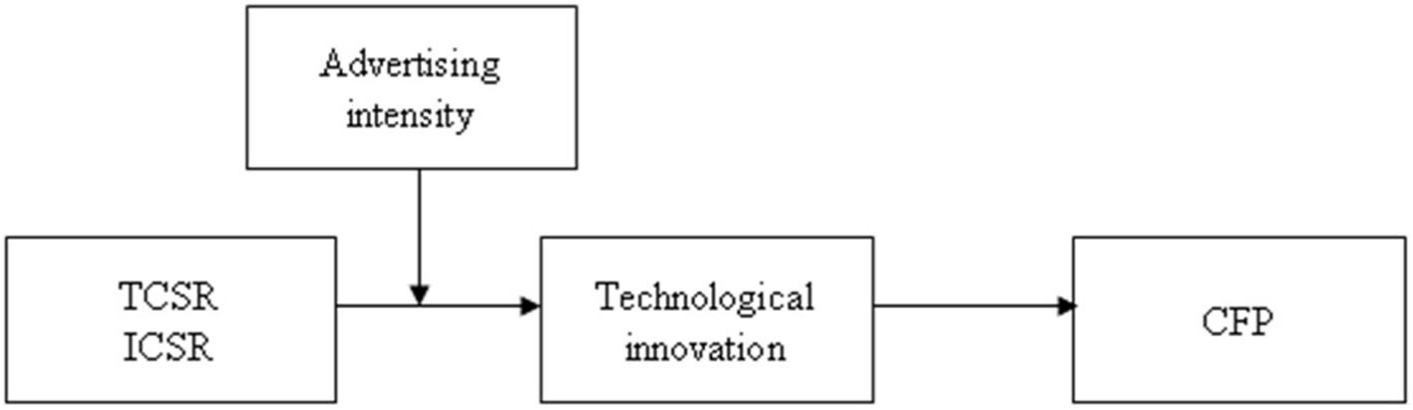
Theoretical model.

As discussed thus far and as shown in Figure 1, we assume that technological innovation mediates the TCSR–CFP relationship and that advertising intensity strengthens or weakens the impact of TCSR on technological innovation. According to these hypotheses, we further infer that the higher the advertising intensity is, the stronger (or weaker) is the indirect impact on the TCSR–CFP relationship through technological innovation; that is, when the advertising intensity increases, the mediating impact on technological innovation in the TCSR–CFP relationship is stronger (or weaker). Thus, we develop two competing hypotheses as follows:

*H6a: The higher the advertising intensity is, the stronger is the mediating effect of technological innovation in the TCSR–CFP relationship*.
*H6b: The higher the advertising intensity is, the weaker is the mediating effect of technological innovation in the TCSR–CFP relationship*.

## Research Design

The overarching goal of our research is to reveal the mechanism through which CSR affects CFP. The regression model we adopt to examine our primary hypothesis about the association between CSR and CFP is as follows:

(1) $$\begin{array}{r} TQ_{t}=\beta_{0}+\beta_{1}TCSR_{t}\left(ICSR_{t}\right)+\beta_{2}Controls_{t}+\varepsilon_{t} \end{array}$$

The dependent variable of our study is CFP. In this study, we measure CFP using the long-term financial performance of firms (i.e., Tobin's Q). The accounting measurements of CFP, such as earnings or returns on asset, focus on retrospective short-term performance, and managers are likely to manipulate those accounting numbers through their discretion in accounting policies (Hillman and Keim, 2001). In contrast, Tobin's Q, which is a proxy for firm valuation, can more effectively measure CFP in the long run because the incremental value of a firm does not only present its current financial position but also reflects the positive reactions of the financial market on the prospects of the firm's intangible assets (Dowell et al., 2000). Therefore, we follow Surroca et al. (2010) to use Tobin's Q as the proxy of CFP. Given the difficulty in obtaining data on the replacement value of assets, we measure Tobin's Q (*TQ*_t_) as the ratio of market value to total assets, computed as the number of outstanding shares multiplied by the closing price at the end of the fiscal year, divided by total assets.

Then, to assess the firms' CSR performance, we use the Reactive–Defensive–Accommodative–Proactive Scale developed by Clarkson (1995) and the stakeholder theory, to construct a model with seven groups of the related stakeholders that benefit from firms' CSR behaviors: creditors, consumers, shareholders, employees, suppliers, the government, and society. For each group, we adopt one or two variables as proxies to conduct a factor analysis. To be more specific, we use the current ratio (*CR*_*t*_), measured as current assets divided by current liabilities, and the inverse of the leverage ratio (*LEV*_*t*_), measured as total assets divided by total liabilities (times negative one) as proxy for the responsibilities taken on for creditors, because a high level of liability is often associated with a high default risk (Cathcart et al., 2020). We use the growth rate of operational expenses and sales revenues (*CSGR*_*t*_ and *IRR*_*t*_) as proxy for responsibilities taken on consumers, where managing operation expenses and sales revenues of firms efficiently can lead customers to obtain value at a lower cost (Chun and Ovchinnikov, 2019). We use the earnings per share (*EPS*_*t*_), measured as the net profit divided by the number of outstanding shares, and the net asset per share (*NAPS*_*t*_), measured as net assets divided by the number of outstanding shares, as proxy for responsibilities taken on shareholders because these measures indicate the actual value, rather than the market price of the shares held by shareholders. We use employee benefits (*EBL*_*t*_), measured as cash paid to (for) employees scaled by sales revenue, as proxy for responsibilities taken by employees. We use operation expenses divided by accounts payables to measure the accounts payable turnover rate (*APTR*_*t*_) as the proxy for responsibilities taken on suppliers because a high level of accounts payable leads to high risks and uncertainty in the cashflow of suppliers (Nam and Uchida, 2019). Considering that the government always has to handle the serious problem of unemployment and tax is an essential source of government revenue, we use jobs created (*ER*_*t*_, measured as the number of employees, scaled by net assets) and tax responsibility (*ATR*_*t*_, measured as tax paid minus tax rebates received, scaled by net assets) as proxy for responsibilities taken on by the government. Last, we use the expenditure on donations (*DIR*_*t*_) as proxy for the responsibilities taken by society because the donation is a channel for firms to help people in society who experience poverty and natural disasters (Wang et al., 2015). To address the concerns about the skewed distribution of variables (Martikainen et al., 1995) and multicollinearity, we standardize the variables included before we conduct factor analysis.

Table 1 reports the outcome of factor analysis. Panel A presents the results of the Kaiser–Meyer–Olkin and Bartlett's tests. The sampling adequacy is 0.527 (significance = 0.000 < 0.05), which is higher than 0.5, showing that factor analysis is appropriate for our model. We then adopt the principal component analysis method to extract seven components. Panel B presents the total variance explained by each component. The total accumulative variance contribution after rotation is 91.376%, which implies the components extracted cover the overall information well. We compute the weight of each component as its variance contribution, divided by the total accumulative variance contribution (91.376%). Further, Panel C presents the rotated component matrix. We define the component proxying each group based on its highest factor loadings. For example, *CR*_*t*_ and *LEV*_*t*_ have the highest two factor loadings in component 1, which implies the score of component 1 measures the performance of CSR regarding creditors. Accordingly, the scores of component 2 to component 7 measure the performance of CSR regarding consumers, shareholders, the government, employees, society, and suppliers. Last, following Mattingly and Berman (2006) and Godfrey et al. (2009), we extract data for the score of each component and define institutional CSR (*ICSR*_*t*_) as the weighted score of component 6 (for society), and technical CSR (*TCSR*_*t*_) as the weighted sum of the remaining six components' scores.

**Table 1:** Factor analysis: measuring firm CSR performance.

**Panel A KMO and Bartlett's tests**							
Kaiser-Meyer-Olkin measure of sampling adequacy				Sampling adequacy			0.527
Bartlett's test of sphericity				Approx. chi-square			56884.995
				df			55.000
				Sig.			0.000
	**Extraction sums of squared loadings**			**Rotation sums of squared loadings**			
	**Total**	**% of variance**	**Cumulative %**	**Total**	**% of variance**	**Cumulative %**	
**Panel B total variance explained**							
1	2.241	20.369	20.369	1.929	17.532	17.532	
2	1.928	17.523	37.892	1.812	16.476	34.008	
3	1.576	14.326	52.219	1.727	15.697	49.704	
4	1.409	12.811	65.029	1.583	14.392	64.096	
5	1.001	9.096	74.125	1.001	9.098	73.194	
6	1.000	9.086	83.211	1.000	9.091	82.285	
7	0.898	8.165	91.376	1.000	9.091	91.376	
	**1**	**2**	**3**	**4**	**5**	**6**	**7**
**Panel C rotated component matrix**							
*CR_*t*_*	**0.974**	−0.007	0.086	−0.016	0.061	−0.003	0.005
*LEV_*t*_*	**0.972**	−0.010	0.087	−0.025	0.078	−0.004	0.002
*IRR_*t*_*	−0.006	**0.949**	0.070	−0.001	−0.002	0.000	0.000
*CSGR_*t*_*	−0.011	**0.948**	0.079	−0.001	−0.022	−0.001	0.000
*EPS_*t*_*	0.034	0.053	**0.929**	0.000	−0.056	0.004	−0.007
*NAPS_*t*_*	0.135	0.096	**0.914**	−0.020	0.004	−0.003	0.002
*EBL_*t*_*	0.119	−0.021	−0.048	0.016	**0.989**	0.002	0.001
*APTR_*t*_*	0.006	0.000	−0.005	0.001	0.001	0.000	**1.000**
*ER_*t*_*	−0.017	0.005	−0.016	**0.888**	0.074	−0.004	−0.001
*ATR_*t*_*	−0.019	−0.007	−0.002	**0.891**	−0.057	0.005	0.002
*DIR_*t*_*	−0.005	−0.001	0.001	0.001	0.002	**1.000**	0.000

*Bold values indicate the highest factor loading of each variable*.

In addition to the variables for CFP and CSR performance, our model includes firm size (*SIZE*_*t*_), firm age (*AGE*_*t*_), state ownership (*STATE*_*t*_), and the specific characteristics (*IT*_*t*_) of the internet technology (IT) industry (i.e., a significant level of intangible assets) as our control variables. Further, large firms have more resources to pursue long-term benefits (Akram et al., 2020). Hence, we measure *SIZE*_*t*_ as the natural logarithm of total assets. Next, mature firms are more experienced in seeking additional investment opportunities, such as CSR activities, to promote their valuation (Wang et al., 2015). Therefore, we measure *AGE*_*t*_ as the time gap between the current year and the foundation year of the firm. Moreover, state-owned firms often underperform in financial markets because of their less effective corporate governance (Lazzarini and Musacchio, 2018). Hence, we compute *STATE*_*t*_ as the number of shares held by the state, divided by the number of total shares. Last, because IT firms are often better priced in the financial market because of their valuable intangible assets (Banker et al., 2019), we create an indicator for the internet technology industry classified based on the *Guidance for Industry Classification of Listed Companies* (2012 revised edition) of the China Securities Regulatory Commission.

To examine the mediating role of innovation, we adopt a regression model for the relationship between CSR performance and innovation. We measure technological innovation with R&D spending (*R*&*D*_*t*_), measured as R&D expenses scaled by total assets. We use Models (1), (2), and (3) to jointly examine whether firms' CSR performance influences their CFP by affecting technological innovation:

(2) $$\begin{array}{r} {R\&D}_{t}=\beta_{0}+\beta_{1}TCSR_{t}\left(ICSR_{t}\right)+\beta_{3}Controls_{t}+\varepsilon_{t} \end{array}$$

(3) $$\begin{array}{r} TQ_{t}=\beta_{0}+\beta_{1}{R\;\&D}_{t}+\beta_{2}TCSR_{t}\left(ICSR_{t}\right)+\beta_{3}Controls_{t}\\ \;+\varepsilon_{t} \end{array}$$

Last, we investigate whether the mediating effects of technological innovation are moderated by advertising intensity. We measure our moderating variable, *ADI*_*t*_, as the sales expense, scaled by sales revenue. To address collinearity concerns, before we interact *ADI*_*t*_ with our variable of interest, *TCSR*_*t*_, we decentralize the data for those two variables. We adopt a structural equation model, shown in Models (4) and (5), to test whether *ADI*_*t*_ moderates the mediation models of technological innovation:

(4) $$\begin{array}{r} {R\&D}_{t}=\beta_{0}+\beta_{1}TCSR_{t}+\beta_{2}ADI_{t}+\beta_{3}TCSR_{t}\times ADI_{t}\\ \;+\beta_{4}Controls_{t}+\varepsilon_{t} \end{array}$$

(5) $$\begin{array}{r} TQ_{t}=\beta_{0}+\beta_{1}{R\;\&D}_{t}+\beta_{2}TCSR_{t}+\beta_{3}ADI_{t}+\beta_{4}TCSR_{t}\\ \times \;ADI_{t}+\beta_{5}Controls_{t}+\varepsilon_{t} \end{array}$$

## Empirical Results

### Sample and Data

Our sample covers the firm-year observations listed in China's A-share market from 2007 to 2018. The starting year is 2007 because data on R&D expenses are available from this year onward. In addition, we follow the prior literature and exclude firms from the financial industry because of the significant differences in their financial characteristics (Cho and Lee, 2019). We obtain all the data from the China Stock Market & Accounting Research Database. Thus, we obtain 13,384 firm-year observations for 2,552 unique firms.

Panel A of Table 2 presents the summary statistics of all the variables included in our study. Overall, the market value of firms in our sample is around 2.454 times the book value of their total assets. Their TCSR performance scores range from −4.509 to 16.885, and their ICSR scores range from −0.003 to 7.678. On average, these firms spend 4.4% of their sales revenue on R&D, and 7.5% on advertising. The average firm age is approximately 3 years, the average state ownership is 3.8%, and 8.6% of the firms in the sample compete in the internet technology industry.

**Table 2:** Descriptive statistics, correlation matrix, and VIF analysis.

**Variable**		**Mean**		**SD**		**Min**		**Median**		**Max**
**Panel A descriptive statistics**										
*TQ_*t*_*		2.454		2.535		0.083		1.837		128.438
*TCSR_*t*_*		0.000		0.379		−4.609		−0.070		16.885
*ICSR_*t*_*		0.000		0.099		−0.033		−0.003		7.678
*R*&*D_*t*_*		0.044		0.061		0.000		0.034		2.516
*ADI_*t*_*		0.075		0.084		0.000		0.047		1.116
*SIZE_*t*_*		22.022		1.286		17.806		21.820		28.509
*AGE_*t*_*		2.717		0.394		0.693		21.820		3.932
*STATE_*t*_*		0.038		0.120		0.000		0.000		0.875
*IT_*t*_*		0.086		0.280		0.000		0.000		1.000
*CR_*t*_*		2.824		4.535		0.075		1.721		190.869
*LEV_*t*_*		3.835		4.666		0.125		2.517		132.956
*IRR_*t*_*		0.509		17.829		−0.949		0.120		1880.750
*CSGR_*t*_*		0.341		5.166		−0.894		0.128		317.029
*EPS_*t*_*		0.668		1.289		−11.055		0.384		31.387
*NAPS_*t*_*		8.587		8.943		−9.652		5.860		199.041
*EBL_*t*_*		0.034		0.025		−0.044		0.028		0.337
*APTR_*t*_*		23.015		1110.664		0.278		6.299		127301.600
*ER_*t*_*		0.000		0.000		0.000		0.000		0.001
*ATR_*t*_*		0.014		0.048		−2.050		0.010		2.429
*DIR_*t*_*		0.000		0.015		0.000		0.000		1.186
	**1**	**2**	**3**	**4**	**5**	**6**	**7**	**8**	**9**	**VIF**
**Panel B correlation matrix**										
*TQ_*t*_*	1.000									
*TCSR_*t*_*	0.157	1.000								1.178
*ICSR_*t*_*	−0.011	0.000	1.000							1.153
*R*&*D_*t*_*	0.211	0.213	−0.008	1.000						1.110
*ADI_*t*_*	0.180	0.155	0.008	0.183	1.000					1.087
*SIZE_*t*_*	−0.423	−0.145	0.029	−0.195	−0.180	1.000				1.078
*AGE_*t*_*	−0.042	−0.107	0.014	−0.089	−0.013	0.218	1.000			1.065
*STATE_*t*_*	−0.069	0.034	0.008	−0.064	−0.091	0.162	−0.039	1.000		1.043
*IT_*t*_*	0.181	0.129	−0.007	0.296	0.109	−0.138	−0.037	−0.038	1.000	1.001

We report the correlation matrix and the results of the variance inflation factor (VIF) tests in Panel B. The correlation matrix shows that TCSR performance and technological innovation are positively related to Tobin's Q, which primarily supports our hypotheses on TCSR. In addition, ICSR negatively relates to Tobin's Q, which implies that the different types of CSR performance may have different effects on CFP. All the correlations are less than 0.500, and the VIF statistics for all variables are about 1, less than the recommended cut-off of 10 (Hair, 2009), which indicates that multicollinearity is not a serious issue in our empirical model.

### Regression Results

To address how CSR performance, innovation, and CFP interact with each other, we first adopt the hierarchical regression method (Baron and Kenny, 1986) to run the regression models for the relationship between CSR and CFP, between CSR and technological innovation, and between CSR and CFP, after controlling for technological innovation. Panel A of Table 3 reports the regressions results for Models (1), (2), and (3), which examine the associations between CSR performance, technological innovation, and CFP, respectively. In Column (1), the estimated coefficient on *TCSR*_*t*_ is positive and significant (β = 0.600, *p* < 0.01), which implies that the TCSR performance is positively related to CFP; thus, H1 is supported. In addition, we find that older firms and IT firms have a better valuation in the financial market, and state ownership does not influence CFP. The estimated coefficient on *TCSR*_*t*_ in Column (2) is 0.026 (*p* < 0.01), suggesting that as firms perform better in TCSR, their spending on R&D increases. Older firms and state-owned firms are found to spend less on R&D than non-state-owned firms, whereas IT firms spend more on R&D than firms in other industries. In Column (3), the estimated coefficient on *R*&*D*_*t*_ is 4.035 (*p* < 0.01), which supports the positive impact of innovation on promoting CFP. After controlling for the effects of *R*&*D*_*t*_, the estimated coefficient of *TCSR*_*t*_ remains significant and positive (β = 0.495, *p* < 0.01), primarily showing that technological innovation plays a partial mediating role in the association between TCSR performance and CFP. Therefore, these results support H3.

**Table 3:** CSR performance and CFP: The mediating effects of innovation.

	**The effects of** ***TCSR***			**The effects of** ***ICSR***		
**Dependent variable:**	**TQ**	**R&D**	**TQ**	**TQ**	**R&D**	**TQ**
	**(1)**	**(2)**	**(3)**	**(4)**	**(5)**	**(6)**
**Panel A regression results**						
*R*&*D_*t*_*			4.035***			4.566***
			(0.342)			(0.339)
*TCSR_*t*_*	0.600***	0.026***	0.495***			
	(0.053)	(0.001)	(0.053)			
*ICSR_*t*_*				0.029	0.000	0.030
				(0.198)	(0.005)	(0.196)
*SIZE_*t*_*	−0.803***	−0.006***	−0.779***	−0.824***	−0.007***	−0.794***
	(0.016)	(0.000)	(0.016)	(0.016)	(0.000)	(0.016)
*AGE_*t*_*	0.389***	−0.006***	0.413***	0.346***	−0.008***	0.382***
	(0.051)	(0.001)	(0.051)	(0.051)	(0.001)	(0.051)
*STATE_*t*_*	0.023	−0.021***	0.110	0.127	−0.017***	0.204
	(0.167)	(0.004)	(0.166)	(0.167)	(0.004)	(0.166)
*IT_*t*_*	1.046***	0.056***	0.821***	1.136***	0.060***	0.864***
	(0.071)	(0.002)	(0.073)	(0.071)	(0.002)	(0.073)
F-statistics	687.59***	433.78***	602.03***	655.40***	347.37***	583.82***
R^2^	0.204	0.140	0.213	0.197	0.115	0.208
Adj.R^2^	0.204	0.139	0.212	0.197	0.115	0.207
	**The effects of** ***TCSR***			**The effects of** ***ICSR***		
**Panel B bootstrap: Sobel-Goodman mediation tests**						
Indirect effects	0.105***			−0.001		
	(0.031)			(0.013)		
Direct effects	0.495***			0.030		
	(0.162)			(0.141)		
Replication	5,000			5,000		

The table presents the regression results of examinations on the relationship between CSR performance and financial performance, and the mediating effects of innovation on this relationship. Panel A presents the regression results. The results in Column 1 (4) reports the association among technological (institutional) CSR performance and financial performance; the results in Column 2 (5) reports the association among technological (institutional) CSR performance and R&D expenses; and the results in Column 3 (6) reports the mediating effects of R&D expenses on the association among technological (institutional) CSR performance and financial performance. Panel B presents the results of Sobel-Goodman mediation tests replicating 5000 times with bootstrap method. Detailed definitions of variables are reported in the Appendix A. Standard errors are reported in brackets. Significance at the 1% level is denoted by ^***^.

Moreover, we adopt the bootstrap resampling method to test the mediating effects of technological innovation, which is suggested to have the strongest test power on mediation effects compared with all other methods, including the hierarchical regression method (MacKinnon et al., 2004). Panel B presents the results of Sobel–Goodman mediation tests replicated 5,000 times with the bootstrap resampling method. We find that while TCSR also positively affects CFP directly (β = 0.495, *p* < 0.01), it also has positive indirect effects on CFP through technological innovation (β = 0.105, *p* < 0.01). The results are consistent with our primary findings for H3.

By contrast, the estimated coefficients on *ICSR*_*t*_ in Columns (4), (5), and (6) are not significant (*p* > 0.10), which together with the nonsignificant results of the bootstrap resampling test, imply that ICSR performance is not related to innovation spending or to CFP. The findings support H4 but do not support H2. A potential explanation is that unlike US firms, under the Chinese system, firms cannot extract direct financial benefits, such as tax benefits, through charitable donations, given that only about 3% of all the charity organizations in China are exempt from tax (Su and He, 2010).

Next, we examine whether the mediating effects of technological innovation are moderated by advertising intensity. Table 4 reports the regression results of tests on the moderating effects of advertising intensity on the association between TCSR performance and technological innovation, and a structural equation for the relationships between TCSR, technological innovation as a mediator moderated by advertising intensity, and CFP. In Column (1), the results show that the interaction term of TCSR and technological innovation (*TCSR*_*t*_ × *ADI*_*t*_) positively relates to technological innovation spending (β = 0.179, *p* < 0.01), suggesting that advertising intensity has positive incremental effects on the positive impact of TCSR on technological innovation. The results support H5a but do not support H5b.

**Table 4:** TCSR and CFP: The mediating effects of innovation moderated by advertising.

**Dependent variable:**	**R&D**	**R&D**	**TQ**
	**(1)**	**(2)**	**(3)**
**Panel A regression results**			
*R*&*D_*t*_*			6.797***
			(0.359)
*TCSR_*t*_*	0.027***	0.036***	0.687***
	(0.001)	(0.001)	(0.060)
*ADI_*t*_*	0.059***	0.082***	3.980***
	(0.006)	(0.006)	(0.270)
*TCSR*_*t*_ × *ADI*_*t*_	0.179***	0.242***	0.519
	(0.017)	(0.018)	(0.757)
*SIZE_*t*_*	−0.005***		
	(0.000)		
*AGE_*t*_*	−0.006***		
	(0.001)		
*STATE_*t*_*	−0.017***		
	(0.004)		
*IT_*t*_*	0.052***		
	(0.002)		
F-statistics	357.54***	407.58***	191.14***
R^2^	0.158	0.084	0.054
Adj.R^2^	0.157	0.083	0.054
**Panel B bootstrap: Moderated mediation tests based on structural**			
**equation model**			
*R*&*D_*mean*−*sd*_*		0.225***	
		(0.052)	
*R*&*D_*mean*_*		0.364***	
		(0.068)	
*R*&*D_*mean*+*sd*_*		0.503***	
		(0.098)	
Replication		5,000	

The table presents the regression results of examinations on the moderating effects of advertising intensity on the mediating role of innovation playing in the relationship between TCSR performance and financial performance. Panel A presents the regression results. The results in Column 1 reports the association among TCSR performance and R&D expenses moderated by advertising expenses, including the control variables; the results in Column 2 reports the first stage of structural equation model for the association among TCSR performance and R&D expenses moderated by advertising expenses; and the results in Column 3 reports the second stage of structural equation model for the mediating effects of R&D expenses moderated by advertising expenses, on the association among TCSR performance and financial performance. Panel B presents the results of structural equation replicating 5000 times with bootstrap method for R&D expenses with the value of mean minus standard deviation, mean, and mean plus standard deviation, respectively. Detailed definitions of variables are reported in the Appendix A. Standard errors are reported in brackets. Significance at the 1% level is denoted by ^***^.

After uncovering the moderating effects of advertising intensity on the positive relationship between TCSR and technological innovation, we follow Edwards and Lambert (2007) and adopt a structural equation to test whether advertising intensity moderates the role of technological innovation as a mediator. In Column (2) of Table 4, the results for the first stage of the structural equation are consistent with our findings in Column (1), where the interaction term is positively associated with technological innovation (β = 0.242, *p* < 0.01). At the second stage of the structural equation, the estimated coefficient of interaction in Column (3) is not significant (β = 0.519, *p* > 0.10), which implies that advertising intensity does not moderate the direct effects of TCSR on CFP. Thus, the results of the two stages of the structural equation jointly support H6a that advertising intensity positively moderates the mediating role of technological innovation in the relationship between TCSR and CFP. Last, we replicate the structural equation 5,000 times using the bootstrap resampling method. The results show that on taking the mean minus one standard deviation, the mean, and the mean plus one standard deviation of sales expenses scaled by sales revenue as the value of *ADI*_*t*_, the mediating effects of technological innovation show a trend of growth from 0.225 (*p* < 0.01) to 0.503 (*p* < 0.01), which is consistent with our primary findings for H6a.

## Conclusion and Discussion

Our study, which combines the stakeholder theory and the knowledge-based view, uncovers that technological innovation is the key mechanism bridging CSR heterogeneity and CFP. Using the main effect model, we also prove that advertising intensity, as a critical market differentiation strategy, plays the role of moderator in the entire relationship between CSR heterogeneity and CFP. Therefore, we reach the following conclusions.

First, by considering the various stakeholder recipients of firms, we differentiate CSR activities into TCSR and ICSR, which refer to socially responsible initiatives that target the primary stakeholders and the secondary stakeholders of a firm, respectively. We argue that TCSR can enhance CFP significantly whereas ICSR engagement may not lead to CFP improvement. TCSR can drive profit gaining through firms' technological innovation; that is, the mediating effect of technological innovation can be valid only in the TCSR–CFP relationship and not in the ICSR–CFP relationship. These results on the nonsignificant impact on the ICSR–CFP relationship may lead to the reconsideration of the relationship between charitable giving and corporate financial goals. Charitable behaviors reflect the “concern-for-others” corporate philosophy, and the purpose of firms' kindness is to acquire and accumulate reputational capital and boost corporate prosperity. Reputational capital is of great value to firms for business development—for instance, to help alleviate resource restraints, create differentiated advantages, improve customer loyalty, and reduce the employee turnover rate. From the other side, philanthropic actions may impair CFP in light of the principal–agent relationship between the manager and the firm. This effect occurs because the firm's generosity may improve only its managers' reputation or translate to their private social capital, and these social advantages generated by charitable behaviors would not be passed on to the firm level and facilitate CFP (Haley, 1991). In this study, our results show that there is a positive but nonsignificant correlation between ICSR and CFP, which may be attributable to the latent negative impact on the correlation between charitable donation behaviors and CFP. Moreover, we argue that it is difficult for firms to apply ICSR strategies directly to their core business competencies, and the result is consistent with the views of Madsen and Rodgers (2015). These authors also suggest that CSR activities targeted to the secondary stakeholders cannot contribute directly to their welfare, and thus, firms cannot obtain any immediate benefits and rewards from these stakeholders.

Second, we proposed two competing hypotheses to test whether the relationship between advertising intensity and TCSR is synergistic or substitutional. We conclude that there is a synergistic interaction between advertising intensity and TCSR, which means advertising can strengthen the linkage between TCSR and technological innovation. This finding confirms the view that a good reputation generated from high advertising intensity reinforces the knowledge exchange between the primary stakeholders and corporations, and consequently drives technological innovation improvement. Moreover, this finding further illustrates that advertising intensity and TCSR have different roles in ensuring product differentiation from competitors and reducing the information asymmetry between goods and customers. In other words, firms' decision-makers need not regard advertising as the only key to creating product varieties and decreasing information asymmetry, and TCSR is another efficient approach to help build technology innovation capabilities. Furthermore, from a long-term perspective, the synergetic relationship between TCSR and advertising intensity emphasizes the importance of prioritizing stakeholders' interests and maintaining stakeholder relationships for long-term business growth. Otherwise, managers would blindly focus on short-term profits and pay too much attention to market strategies such that they myopically overlook stakeholders' requirements.

Third, advertising intensity also moderates the mediated relationship of TCSR with CFP such that higher advertising intensity strengthens the mediating effect of technological innovation on the TCSR–CFP relationship. This finding reveals that firms with large advertising investments can maximize their profits not only by relying on the customer loyalty generated through their advertising strategy, but also by focusing on their main value creation mechanism in business. This study recommends that managers in these firms should avoid depending on high advertising intensity and indulging in earning quick profits. Therefore, firms with advertising advantages should be outstandingly competent in the aspects of product prices, product quality, and innovation. Only their outperformance in both advertising and technological innovation will enable businesses to thrive in a sustainable manner.

## Implications and Limitations

This study has several theoretical and practical implications. Theoretically, first, the theoretical model incorporates the knowledge-based view into the stakeholder theory, which provides the causal relationship between CSR and CFP explained by the stakeholder theory with additional evidence that superior CSR performance can enhance CFP by promoting technological innovation. Second, this study responds to the calls in the literature for further research to identify the mechanism underlying the CSR–CFP relationship in the specific context. In this study, we include technological innovation and advertising intensity as contingent factors in examining the CSR–CFP relationship. Third, we distinguish CSR into TCSR and ICSR activities, which echoes the prior suggestion that since CSR involves heterogeneous activities, the different factors in CSR should not be integrated into a single variable.

This study also has some managerial implications. First, we provide robust evidence that TCSR can help achieve firms' financial goals through technological innovation, which could be the clue for firms' decision-makers to integrate TCSR strategies with the core business competency for profit maximization. In a rebuttal to those who have suggested that participating in CSR activities is a diversion of limited resources and managerial efforts from the core business, this study recommends that investment in TCSR activities should be regarded as a valuable capital investment rather than an operational cost. Second, combining the CSR strategy with other traditional core strategies, such as technological innovation and advertising market strategy in order to impel innovation strategy, would result in significant efficiency. In addition, given the synergistic interaction between TCSR and advertising intensity, to prevent a shortsighted focus on profit-making, firms with high advertising intensity should concentrate on building an innovative and responsibility-oriented corporate culture. Meanwhile, efforts should be made to strengthen the synergistic effect of the two strategies in promoting technological innovation, which would allow firms to run their operations in a benign development mode. Further, an aspect that cannot be neglected is the supportive policies provided by institutions. We suggest the government should encourage firms to participate in CSR activities and become involved in creative activities with adequate corresponding policy assurance. Last, from the knowledge-based view, firms need to raise awareness of CSR among their different stakeholder groups and enhance knowledge exchange in their knowledge networks through their CSR communication with their stakeholders (or stakeholder groups).

This study does have some limitations. First, it relies entirely on secondary data, and although archival databases are objective and reliable, these do not provide access to the perceptions and other subjective factors that influence managerial decisions. Second, given that R&D expenditure is a voluntary disclosure rather than mandatory in the annual reports of listed firms in China, we cannot obtain R&D information for every listed company, which may lead to some deviations. Third, we fail to disentangle the technological innovation concept into different dimensions. Thus, in a future study, we intend to consider different types of innovation, such as exploratory innovation and exploitative innovation to conduct a more comprehensive, in-depth analysis.

## Data Availability Statement

Publicly available datasets were analyzed in this study. This data can be found here: China Stock Market & Accounting Research Database (CSMAR), available at https://www.gtarsc.com.

## Author Contributions

MN presented the idea and wrote the theoretical part of the manuscript. WM built the structure of the article, wrote empirical analysis of the manuscript, and revamped the parts requested during peer review throughout the whole manuscript. Both authors contributed to the article and approved the submitted version.

## Funding

This work was supported by National Natural Science Foundation of China (71732004).

## Conflict of Interest

The authors declare that the research was conducted in the absence of any commercial or financial relationships that could be construed as a potential conflict of interest.

## Publisher's Note

All claims expressed in this article are solely those of the authors and do not necessarily represent those of their affiliated organizations, or those of the publisher, the editors and the reviewers. Any product that may be evaluated in this article, or claim that may be made by its manufacturer, is not guaranteed or endorsed by the publisher.

## Appendix I

**Table A1:** Variables definitions.

**Variable**		**Definition**
*TCSR_t_*		Technical CSR performance, measured as the weighted sum of scores of components proxying responsibilities to creditors, consumers, shareholders, employees, suppliers, and government, extracted from factor analysis. Details are introduced in Section 3.2
*ICSR_t_*		Institutional CSR performance, measured as the weighted score of the component proxying responsibilities to society. Details are introduced in Section 3.2
*TQ_t_*		Tobin's Q, measured as the number of outstanding shares multiplies annual closing price, divided by total asset.
*R&D_t_*		R&D expense, scaled by sales revenue.
*ADI_t_*		Advertising level, measured as sales expenses scaled by sales revenue.
*SIZE_t_*		Firm size, measured as the natural logarithm of total asset.
*AGE_t_*		Firm age, measured as current year minus the foundation year of the firm.
*STATE_t_*		State ownership, measured as the number of shares state held, divided by the number of total shares.
*IT_t_*		Indicator variable, equals one if the firm competes in internet technology industry, classified based on the Guidance for Industry Classification of Listed Companies (2012 revised edition) of CSRC.
**Variable**	**Related parties**	**Definition**
**The variables used in factor analysis to compute CSR performance score**		
*CR_t_*	Creditors	Current ratio, computed as current asset divided by current liability
*LEV_t_*		Inverse of leverage ratio, computed as total asset divided by total liability
*CSGR_t_*	Consumers	Growth rate of operation expense, computed as the changes in operation expense, divided by lagged operation expense.
*IRR_t_*		Growth rate of sales revenue, computed as the changes in sales revenue, divided by lagged sales revenue.
*EPS_t_*	Shareholders	Earnings per share, computed as net profit divided by the number of outstanding shares.
*NAPS_t_*		Net asset per share, computed as net asset divided by the number of outstanding shares.
*EBL_t_*	Employees	Employee benefits, measured as cash paid to employees and cash paid for employees, scaled by sales revenue.
*APTR_t_*	Suppliers	Account payable turnover rate, computed as operation expenses, divided by account payable.
*ER_t_*	Government	Jobs created, measured as the number of employees, scaled by net asset.
*ATR_t_*		Tax responsibility, measured as tax paid minus Tax rebates received, scaled by net asset.
*DIR_t_*	Society	Donations, scaled by sales revenue.
